# Physicochemical Properties of Soft and Hard-type Rice Flour According to Moisture Content and High Hydrostatic Pressure Treatment

**DOI:** 10.3390/foods12010227

**Published:** 2023-01-03

**Authors:** Jeong-Hyun Seo, Yeon-Jae Jo, Youn-Ri Lee, Junsoo Lee, Heon-Sang Jeong

**Affiliations:** 1 Department of Food Science and Biotechnology, Chungbuk National University, Cheongju 28644, Republic of Korea; 2 Department of Food and Nutrition, Daejeon Health Sciences College, Daejeon 34504, Republic of Korea

**Keywords:** rice flour, ultra high pressure, physical properties, gelatinization

## Abstract

This study investigated the effects of high hydrostatic pressure (HHP) treatment on the physicochemical properties of rice flour according to its moisture levels in order to develop new materials for processed rice foods. The rice varieties used were the Samkwang variety (normal and hard type) and the Shingil variety (processing and soft type). The moisture content of the rice flour was adjusted to 35–55% and it was treated with the HHP treatment at 400–600 MPa. The water absorption capacity, solubility, and swelling power of the rice flour increased as the moisture levels and pressure increased. The 600 MPa enzymatic hydrolysis-treated rice flour showed similar results to the heat-treated rice flour. Scanning electron microscopy showed few cavities, resulting in a dense structure. X-ray diffraction confirmed that the 23° peak, which indicates the degree of gelatinization, decreased with increasing moisture levels and pressure. The HHP treatment of the rice flour changed its physicochemical properties according to the moisture levels and pressure applied. These results can provide important information on the development and production of various foods.

## 1. Introduction

The high hydrostatic pressure (HHP) treatment is a method of delivering high pressure by sealing liquid or solid food in a flexible container and placing it in a device containing water [1]. HHP treatment can sterilize microorganisms, change and reverse enzyme reaction rates, result in irreversible inactivation, improve tissues, and gel proteins and starches, depending on the applied pressure [2]. Studies on rice starch have reported changes in the physical properties and gelatinization properties of starch gelatinized by heat treatments. The HHP treatment of starch at a pressure of over 400 MPa is a form of non-thermal gelatinization [3] that minimizes the leaching of amylose from starch granules and suppresses the swelling of the granules.

Rice (Oryza sativa) is classified into the Indica and Japonica species, and its structure is comprised of the rice husk, pericarp, spermoderm, aleurone layer, endosperm, and embryo [4]. Rice is mostly comprised of starch and contains protein, fat, dietary fiber, B vitamins, and minerals. Most rice is consumed as a staple food as well as rice cakes, confectionery, beverages, and alcoholic beverages [5]. There has also been a focus on the development of promising rice varieties for various purposes; 251 rice varieties developed by the Rural Development Administration in Korea are listed in the national list of 2015 [6]. It has been reported that starch is gelatinized when treated with HHP at pressures above 400 MPa. However, processed rice varieties may exhibit different characteristics due to differences in their physicochemical characteristics, starch form, and gelatinization characteristics, and it is expected that new characteristics can be confirmed through research and applied to products. Therefore, in this study, in order to confirm the physicochemical characteristics of starch from different rice varieties, the pressure and moisture content were adjusted to confirm the physicochemical characteristics of and to provide basic data on different varieties of rice flour.

## 2. Materials and Methods

### 2.1. Preparation of Rice Flour Samples

The Samkwang (normal rice) and Shingil (processing type rice) rice varieties were provided by the Rural Development Administration in Korea. The Samkwang variety was wet-milled, and the Shingil variety was dry-milled at the Nongshim Flour Mills (Nongshim Flour Mills Co., Ltd., Asan, Republic of Korea) using an air mill (MCM-3, Nara Machinery Co., Ltd., Tokyo, Japan), and whole milled rice flour was used. The rice flour was mixed with distilled water to obtain a final moisture content of 35%, 45%, and 55%. The rice flour samples, with the adjusted moisture content, were vacuum-packed in polyethylene film (6 × 16 cm).

### 2.2. High Hydrostatic Pressure (HHP) Treatment

The rice flour that was vacuum-packed in polyethylene films was subjected to HPP treatment using a high hydrostatic pressure treatment machine (CIP-process controller, Ilsin Autoclave Inc., Daejeon, Republic of Korea) at 400, 500, and 600 MPa for 10 min. The temperature of the pressure chamber was maintained at 25 °C. An HHP treatment machine is a reactor that applies isostatic pressure, using water as the pressure medium without the use of heat or gas. It is comprised of a high-pressure vessel, high-pressure pump, reservoir tank, safety device, alarm system, and control system.

### 2.3. Heat Treatment

The rice flour suspension (rice flour content: 15%) was subjected to heat treatment by incubation in boiling water (100 °C) for 30 min. The heat-treated rice flour was completely gelatinized and used as a control. HHP treatment samples were analyzed in comparison with those subjected to heat treatment.

### 2.4. Water Absorption Index (WAI)

The WAI was measured using the method proposed by Medcalf [7], with some modifications. The HHP samples (2 g, dry basis) were placed in 50 mL centrifuge tubes. Then, 30 mL of distilled water was added and the mixture was allowed to react for 30 min with stirring every 5 min. The samples were centrifuged (MF-300, Hanil Science Inc., Gimpo, Republic of Korea) at 3000 for 10 min and the supernatant was removed. The weight of the precipitate was measured to determine its water absorption capacity.

### 2.5. Solubility and Swelling Power

The solubility and swelling power were measured using a method modified from Medcalf [7]. The HHP samples (2 g, dry basis) were placed in 50 mL centrifuge tubes. Next, 30 mL of distilled water was added, and the mixture was allowed to react for 30 min with stirring every 5 min. The samples were centrifuged (MF-300, Hanil Science Inc., Gimpo, Republic of Korea) at 3000 rpm for 10 min. The supernatant was poured into a constant-weight dry aluminum dish and dried at 105 °C. The weight of the dried product was measured to calculate the solubility, and the swelling power was calculated using the weight of the precipitate and the calculated solubility.

### 2.6. Scanning Electron Microscopy (SEM)

SEM was performed using a method described by Jin [8], with modifications. HHP-treated samples were dried using a freeze dryer (FD8508, Ilsin Autoclave Inc., Daejeon, Republic of Korea) and pulverized to a 100 mesh-size using a mortar. The powdered sample was adhered to the observation side using double-sided tape and coated with gold. Images were obtained at a magnification of 2000× using a scanning electron microscope (ULTRA PLUS, Carl Zeiss AG, Oberkochen, Germany).

### 2.7. X-ray Diffraction

X-ray diffraction was measured using a modified method described by Lee et al. [9]. The HHP-treated samples were dried using a freeze dryer (FD8508, Ilsin Autoclave Inc., Daejeon, Korea) and pulverized to a 100 mesh-size using a mortar. The samples were analyzed using an X-ray diffractometer (D/MAX-1200, Rigaku, Co., Tokyo Japan) under the following conditions: Cu-Kα filter: Ni, scanning speed: 5.0°/min, diffraction angle (2θ): 5–40°.

### 2.8. α-Amylase hydrolysis

α-Amylase hydrolysis was measured using the method described by Xue et al. [10], with some modifications. A suspension was prepared by adding 2 mL of 0.2 M phosphate buffer (pH 6.9) to 50 mg (dry basis) of each sample. For the blank, distilled water was used instead of the sample. α-amylase (0.5 mL, 36 U/mL) was added to the suspension and incubated at 37 °C for 2 h, then centrifuged (MF-300, Hanil Science Inc., Gimpo, Korea) at 3000 rpm for 10 min. DNS reagent (0.2 mL) was added to 0.1 mL of the supernatant, reacted at 100 °C in boiling water for 5 min, and then cooled rapidly by immersion in cold water. It was then diluted with 0.9 mL of distilled water and the absorbance was measured at 550 nm with a UV spectrophotometer (Epoch microplate spectrophotometer, Biotek Instruments, VT, USA). The obtained value was quantified using the maltose standard.

### 2.9. Rapid Viscosity Analysis (RVA)

Pasting properties were evaluated using the approved AACC [11] method using a rapid visco analyzer (RVA TecMaster, Perten Instrument, MA, USA). The sample (3 g) was dispersed in distilled water (25 mL); it was then equilibrated at 50 °C for 1 min. The samples were heated to 95 °C at a heating rate of 12 °C/min and maintained for 2 min and 30 s. Then, the mixture was cooled to 50 °C at a rate of 12 °C/min and the viscosity was measured while maintaining for 2 min. The peak viscosity, trough, final viscosity, breakdown, setback, gelatinization time, and gelatinization temperature were calculated from the RVA viscogram. The gelatinization viscosity unit is expressed as a rapid viscosity unit (RVU).

### 2.10. Statistical Analysis

All analyses were repeated three times and expressed as the mean ± standard deviation. Statistical analyses were performed using the SPSS statistical program (Statistical Package for the Social Science, Ver. 12.0 SPSS Inc., Chicago, IL, USA) to calculate the mean and standard deviation of each treatment group. Significance was tested at the 5% level using Duncan’s multiple range test.

## 3. Results and Discussion

### 3.1. Water Absorption Index

**Figure 1:**
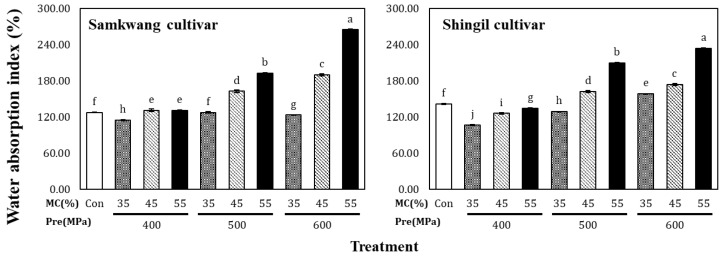
Water absorption capacity of high hydrostatic pressure treated rice flour at different moisture levels. Con: Control, MC: Moisture content of rice flour. Different small letters in the same items indicate a significant result as determined by Duncan’s range test (*p* < 0.05), according to the treatment method.

The water absorption index of the HHP-treated rice flour is shown in Figure 1. The Samkwang variety had a water absorption capacity of 127.56 ± 0.57% in the control, which increased from 114.81 ± 0.56% to 265.14 ± 0.87% as the moisture content and pressure was increased. The Shingil variety had a water absorption capacity of 141.90 ± 0.86% in the control, and for the HHP-treated rice flour, it ranged between 107.03 ± 0.99% and 234.38 ± 0.99%. The rice flour treated at 400 MPa showed a lower water absorption capacity than the control. In the control group, the Shingil variety showed a higher water absorption capacity than the Samkwang variety, which is explained by the difference in milling methods. In general, dry-milled rice flour has a higher content of damaged starch than wet-milled flour [12]. Damaged starch is one of the main factors that increases the water absorption capacity of rice flour. In the case of gelatinized rice flour, the Shingil variety showed a low water absorption capacity. The Shingil variety has a high amylose content, and when the amylose content is high, the swelling of starch is suppressed; this likely influenced the water absorption capacity of the Shingil variety. Moisture is an essential element in starch gelatinization, and it has been reported that HHP treatment can gelatinize starch at 400 MPa [13]. The degree of gelatinization of the starch increased as the moisture content and pressure increased; therefore, it is likely that the water absorption capacity of the rice flour increased because of the gelatinized starch. These results were also consistent with the results from a study by Oh et al. [14], who treated a rice starch suspension at 600 MPa.

### 3.2. Solubility and Swelling Power

**Figure 2:**
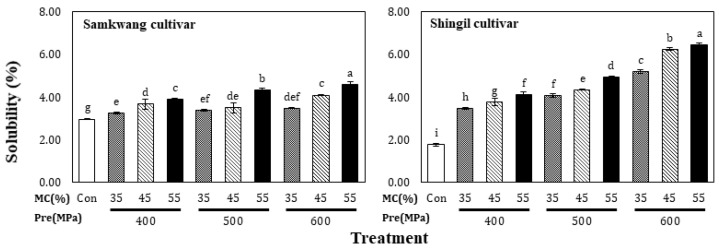
Solubility of high hydrostatic pressure-treated rice flour at different moisture levels. Con: Control, MC: Moisture content of rice flour. Different small letters in the same items indicate a significant result as determined by Duncan’s range test (*p* < 0.05) according to the treatment method.

**Figure 3:**
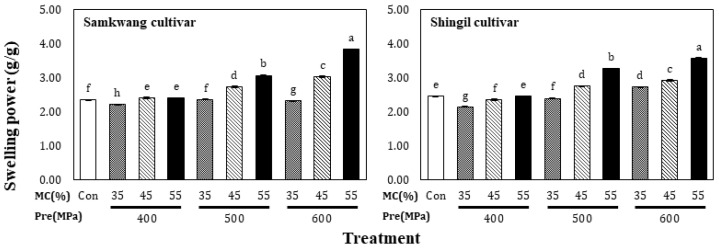
Swelling power of high hydrostatic pressure-treated rice flour at different moisture levels. Con: Control, MC: Moisture content of rice flour. Different small letters in the same items indicate a significant result as determined by Duncan’s range test (*p* < 0.05), according to the treatment method.

The solubility of HHP-treated rice flour is shown in Figure 2. The solubility of the Samkwang variety was 2.96 ± 0.03% in the control and increased from 3.25 ± 0.05% to 4.56 ± 0.12 in the HHP-treated rice flour. The solubility of the Shingil variety was 1.76 ± 0.06% in the control and increased from 3.45 ± 0.05% to 6.42 ± 0.11% with the HHP treatment. The solubility of the Shingil variety was higher than that of the normal Samkwang rice variety. This seems to be the difference between hard-type and soft-type rice. Amylopectin and amylose in the granules are eluted as the starch granules swell and the micelle structure collapses [15]. The eluted starch was combined with water to form a colloidal solution, which greatly increased its solubility. The solubility of the HHP-treated rice flour increased as the moisture content and pressure increased, which is probably because the solubility increased; it has been shown that gelatinizing starch under high pressure can partially decompose the starch granules [16]. Therefore, it is thought that the solubility increased during the HHP treatment, despite the low moisture content. The swelling power of the HHP-treated rice flour is shown in Figure 3; in the Samkwang variety, it was 2.35 ± 0.01 in the control and ranged from 2.22 ± 0.01 to 3.83 ± 0.00 with the HHP treatment. In the Shingil variety, the swelling power was 2.46 ± 0.01 in the control and ranged from 2.14 ± 0.01 to 3.57 ± 0.01 in HHP-treated rice flour. Samkwang, the normal rice variety, showed a lower swelling power than the control at a moisture content of 35% and 400 MPa treatment. The swelling power of the Shingil was lower than that of the control at 400 MPa, regardless of the moisture content. The results were consistent with those obtained for the water absorption capacity, and the swelling power was higher than that of the normal Samkwang rice variety. Swelling power refers to the magnitude of the interactions between starch chains in the amorphous and crystalline regions [17]. The interaction of the Shingil variety used for processing was higher than that of the normal Samkwang rice variety.

### 3.3. Scanning Electron Microscopy (SEM)

**Figure 4:**
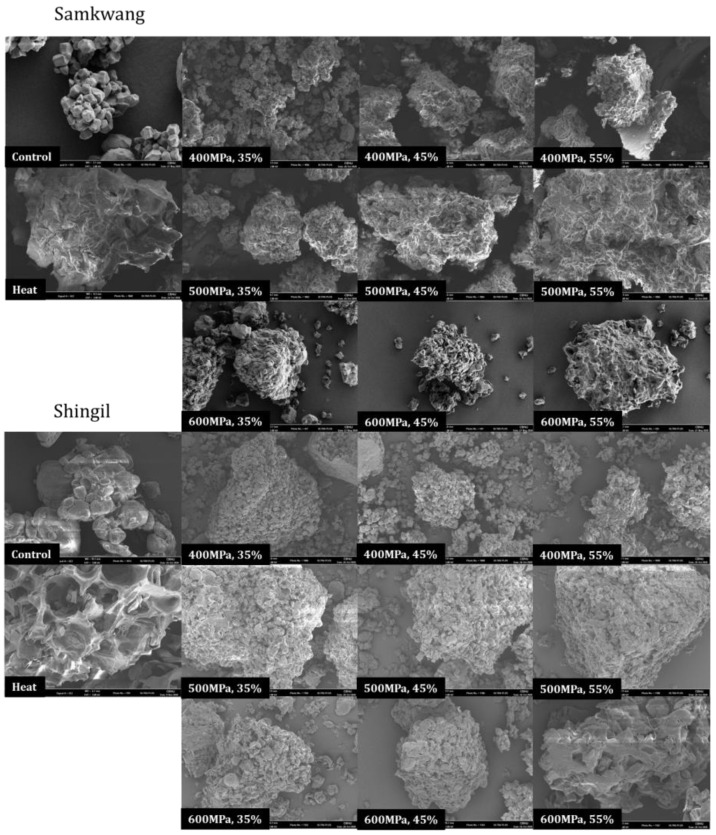
Scanning electron microscopy (2000×) of Samkwang and Shingil variety rice flour at different high hydrostatic pressure treatments and moisture content. 35–55%: Moisture content (%), 400–600 MPa: Pressure.

The SEM images of rice flour subjected to different treatments are shown in Figure 4. Rice starch exhibits a polyhedral shape, and the shape of the starch granules varies depending on the botanical source. In the case of the Samkwang variety, it has a polygonal structure and is observed to have the same structure as general rice starch—but in the case of the processed Shingil variety, it is observed to have a circular shape similar to wheat starch [18]. Gelatinization of the heat-treated rice flour resulted in the micellar structure of the starch collapsing and swelling of the granules. When the moisture content and pressure were low, such as during the 35% moisture content 400 MPa treatment, it could be seen that the starch granules were compressed and that the structure became dense. It was confirmed that the micellar structure of the starch collapsed similarly to during heat treatments as the moisture content and pressure increased—for example, during the treatment with a 55% moisture content at 600 MPa. Rice flour gelatinized by HHP treatment showed smaller starch particles and fewer voids compared to the heat treatment.

### 3.4. X-ray Diffraction

**Figure 5:**
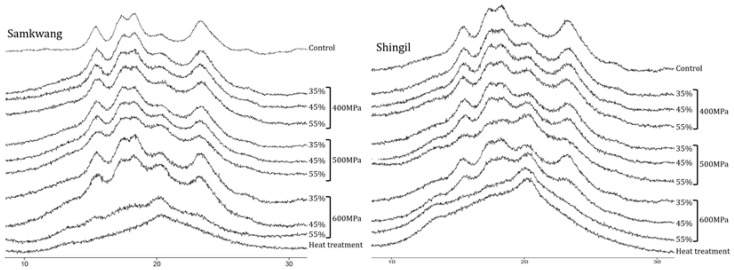
X-ray diffraction pattern of high hydrostatic pressure-treated rice flour at different moisture levels. 35–55%: Moisture content (%), 400–600 MPa: Pressure.

The X-ray diffraction patterns of the HHP-treated rice flours are shown in Figure 5. The Samkwang variety in the control showed strong diffraction peaks at 15.22°, 17.22°, 18.10°, and 23.06°. The Shingil variety showed strong diffraction peaks at 15.22°, 17.06°, 18.08°, and 22.98°. This is the typical diffraction pattern of cereal starch and has the same pattern as type A starch, which shows strong diffraction peaks at 15°, 17°, 18°, and 23° [19]. The heat-treated rice flour lost its crystallinity due to the collapse of the starch granules; hence, no diffraction peaks appeared. Rice flour treated with 55% moisture content at 600 MPa showed a similar pattern to that subjected to heat treatment, regardless of the variety. Among the different X-ray diffraction patterns, the 23° peak reduction rate is an index [20] that determines the gelatinization of starch, and the results of the starch gelatinization based on the peak reduction rate (data not shown) and the enzyme hydrolysis rate showed a tendency to agree with each other. In the case of Samkwang and Shingil rice flour treated with a 35–45% moisture content at 400 MPa, the peak intensity was higher than that of the control because the crystallinity increased as the starch granules were compressed by pressure—as can be seen from the results of SEM. These results show the same results as those of [18,19], which reported that the peak intensity of the x-ray diffraction diagram can appear high as the crystallinity increases.

### 3.5. α-Amylase Hydrolysis

**Figure 6:**
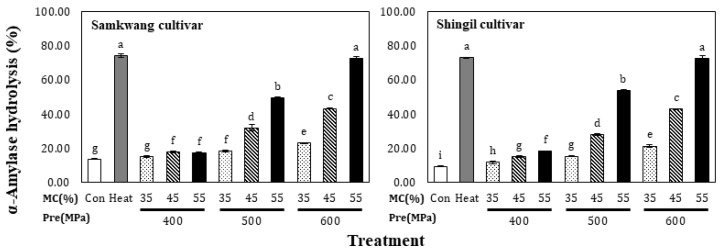
α-Amylase hydrolysis of high hydrostatic pressure-treated rice flour at different moisture levels. Con: Control, Heat: Heat treatment, MC: Moisture content of rice flour. Different small letters in the same items indicate a significant difference as determined by Duncan’s range test (*p* < 0.05), according to the treatment method.

The α-amylase hydrolysis of the HHP-treated rice flour is shown in Figure 6. The Samkwang and Shingil variety control groups showed 13.47 ± 0.11% and 9.05 ± 0.17% α-amylase hydrolysis, respectively. For heat-treated rice flour, the Samkwang and Shingil varieties showed values of 74.02 ± 1.02% and 73.00 ± 0.30%, respectively. In the case of HHP-treated rice flour, depending on the moisture content and increase in pressure, the values for the Samkwang variety ranged from 14.82 ± 0.73% to 72.56 ± 0.88%, and that for the Shingil variety from 11.47 ± 0.95% to 72.39 ± 1.51%. At a 55% moisture content and 600 MPa treatment, both varieties showed a hydrolysis rate similar to that of the heat treatment. The hydrolysis rate tended to be low when the moisture content was low, even with treatment at 600 MPa. This implies that the rice flour can be sufficiently gelatinized when subjected to HHP treatment at a moisture content of 55% and a pressure over 600 MPa. These results suggest that type B starch (raw starch) is not degraded by α-amylase, but when converted to α-starch (gelatinized starch) by heat treatment and HHP treatment, the micellar structure of the starch granules collapses with gelatinization and can be easily digested. This showed the same result as [21] reported, showing that it can be done.

### 3.6. Rapid Viscosity Analysis (RVA)

The pasting properties of the HHP-treated rice flour are shown in Table 1. The peak viscosity, trough, and final viscosity of the Samkwang variety was the highest in the control, at 237.83 ± 0.33, 141.13 ± 0.21, and 257.08 ± 0.42 RVU, respectively. For the Shingil variety, the values were 94.54 ± 0.38, 60.04 ± 0.04, and 136.67 ± 0.17 RVU, respectively. The setback was the highest in the Shingil variety, with 42.12 ± 0.12 RVU, and had a high correlation with degradation. The gelatinization temperatures were 69.98 ± 0.43 and 87.45 ± 0.35 for the Samkwang and Shingil varieties, respectively. The Shingil variety had the highest gelatinization temperature. The peak viscosity, trough, final viscosity, and breakdown tended to decrease and the setback tended to increase when the moisture content and pressure were increased. The pasting properties of the HHP-treated rice flour were higher than those of heat-treated rice flour. According to the SEM results (Figure 4), the granular structure of the heat-treated sample completely collapsed, but the HHP-treated sample did not. Additionally, according to the results of the X-ray pattern (Figure 5) and α-amylase hydrolysis (Figure 6), it can be seen that the 55% moisture content and 600 MPa-treated sample had a similar level of gelatinization to the heat-treated sample. Therefore, the HHP treatment suppressed the swelling of the granules and gelatinized them at the same time. Due to these structural differences, the HHP sample showed a higher peak viscosity, trough, and final viscosity and a lower setback than the heat-treated sample. In the case of the Shingil variety, the RVA and pasting properties were altered because the amylose content was high. According to the results reported by Lee et al. [9], the Goami4 variety, which has an amylose content similar to that of the Shingil variety, also showed a high gelatinization temperature and low viscosity, similar to the results of this study. Starch with a high amylose content suppresses swelling; therefore, the size of the granules was small and the maximum viscosity was low [22,23].

## 4. Conclusions

This study investigated the effects of HHP treatment on the physicochemical properties of rice flour according to its moisture content to develop new materials for processed rice foods. The water absorption capacity, solubility, and swelling power of rice flour, according to the moisture content and HHP treatment, tended to increase with increases in moisture content and pressure. The 55% moisture content and 600 MPa-treated rice flour subjected to enzymatic hydrolysis showed similar results as those subjected to heat treatment. Few cavities were observed in the microstructure (SEM), resulting in a dense structure when the moisture content or pressure was low, and the starch granules collapsed and gelatinized as the moisture content and pressure increased. It was confirmed that the 23° peak, indicating the degree of gelatinization, decreased with increasing moisture content and pressure. The gelatinization temperature of the Shingil variety was significantly higher than that of the Samkwang variety, and its viscosity was low. The physicochemical properties of the HHP-treated rice flour changed according to the moisture levels and pressure. These results provide important information on the development and production of various foods.

## Figures and Tables

**Table 1:** Rapid viscosity analysis (RVA) and pasting properties of high hydrostatic pressure-treated rice flour at different moisture levels.

Variety	Pressure (MPa)	Moisture Content (%)	RVU					Peak Time (min)	Pasting Temperature (°C)
			Peak Viscosity	Trough	Final Viscosity	Breakdown	Setback		
*Sam* *kwang*	Control		237.83 ± 0.33 ^a^	147.13 ± 0.21 ^a b^	257.08 ± 0.42 ^a^	90.71 ± 0.54 ^a^	19.25 ± 0.75 ^f g^	6.37 ± 0.03 ^a b^	69.98 ± 0.43 ^a^
	Heat treatment		156.33 ± 1.00 ^i^	107.35 ± 0.90 ^h^	199.39 ± 0.53 ^i^	48.98 ± 0.10 ^f^	43.05 ± 0.47 ^a^	6.27 ± 0.00 ^b^	63.90 ± 0.75 ^d^
	400	35	236.46 ± 1.38 ^a^	148.79 ± 0.96 ^a^	255.66 ± 1.51 ^b^	87.46 ± 0.29 ^b^	19.20 ± 0.13 ^f g^	6.33 ± 0.00 ^a b^	69.65 ± 0.00 ^a b^
		45	234.17 ± 0.75 ^b^	145.13 ± 0.21 ^b c^	253.42 ± 0.50 ^c^	89.04 ± 0.96 ^a b^	19.25 ± 0.25 ^f g^	6.30 ± 0.03 ^a b^	69.70 ± 0.05 ^a^
		55	232.58 ± 0.58 ^c^	143.29 ± 1.63 ^c^	253.42 ± 0.33 ^c^	89.29 ± 1.04 ^a b^	20.83 ± 0.25 ^e^	6.33 ± 0.00 ^a b^	69.28 ± 0.32 ^a b^
	500	35	237.17 ± 0.08 ^a^	146.25 ± 0.33 ^b^	255.92 ± 0.00 ^b^	90.92 ± 0.25 ^a^	18.75 ± 0.08 ^g^	6.30 ± 0.03 ^a b^	69.30 ± 0.35 ^a b^
		45	222.63 ± 1.20 ^e^	138.23 ± 0.10 ^e^	242.96 ± 0.54 ^e^	84.40 ± 1.10 ^c^	20.33 ± 0.67 ^e f^	6.46 ± 0.01 ^a^	68.43 ± 0.33 ^c^
		55	215.08 ± 0.25 ^f^	131.17 ± 0.75 ^f^	237.83 ± 0.75 ^g^	83.92 ± 1.00 ^c^	22.75 ± 0.50 ^d^	6.37 ± 0.03 ^a b^	69.28 ± 0.43 ^a b^
	600	35	228.50 ± 0.42 ^d^	140.92 ± 0.00 ^d^	248.67 ± 0.25 ^d^	87.58 ± 0.42 ^b^	20.17 ± 0.17 ^e f^	6.37 ± 0.03 ^a b^	68.80 ± 0.05 ^b c^
		45	209.84 ± 1.01 ^g^	129.71 ± 1.71 ^f g^	240.36 ± 0.55 ^f^	80.13 ± 0.70 ^d^	30.53 ± 1.56 ^c^	6.33 ± 0.27 ^a b^	69.15 ± 0.50 ^a b c^
		55	193.43 ± 0.35 ^h^	127.68 ± 2.84 ^g^	228.20 ± 0.71 ^h^	65.75 ± 2.50 ^e^	34.77 ± 1.06 ^b^	6.37 ± 0.03 ^a b^	69.55 ± 0.85 ^a b^
*Shingil*	Control		94.54 ± 0.38 ^c^	60.04 ± 0.04 ^c^	136.67 ± 0.17 ^a^	34.50 ± 0.42 ^a^	42.12 ± 0.21 ^c^	6.13 ± 0.03 ^a^	87.45 ± 0.35 ^c^
	Heat treatment		58.67 ± 0.15 ^h^	42.71 ± 0.10 ^g^	110.53 ± 0.05 ^i^	15.95 ± 0.25 ^f^	51.86 ± 0.20 ^a^	5.83 ± 0.10 ^b^	88.38 ± 0.17 ^b^
	400	35	94.89 ± 0.19 ^b c^	60.58 ± 0.33 ^b^	135.81 ± 0.21 ^c^	34.31 ± 0.53 ^a^	40.92 ± 0.40 ^d e^	6.14 ± 0.04 ^a^	88.45 ± 0.05 ^b^
		45	95.58 ± 0.26 ^a^	60.83 ± 0.00 ^b^	135.98 ± 0.07 ^b c^	34.74 ± 0.26 ^a^	40.41 ± 0.32 ^e^	6.15 ± 0.02 ^a^	88.75 ± 0.65 ^a b^
		55	95.03 ± 0.49 ^b^	60.56 ± 0.28 ^b^	136.13 ± 0.15 ^b^	34.47 ± 0.21 ^a^	41.10 ± 0.63 ^d^	6.15 ± 0.02 ^a^	87.93 ± 0.98 ^b c^
	500	35	95.47 ± 0.25 ^a^	60.83 ± 0.00 ^b^	135.78 ± 0.20 ^c^	34.63 ± 0.25 ^a^	40.32 ± 0.45 ^e^	6.14 ± 0.01 ^a^	88.55 ± 0.00 ^b^
		45	90.35 ± 0.20 ^d^	62.25 ± 0.25 ^a^	132.27 ± 0.19 ^d^	28.10 ± 0.05 ^c^	41.93 ± 0.39 ^c^	6.13 ± 0.00 ^a^	89.03 ± 0.27 ^a^
		55	89.12 ± 0.10 ^e^	62.25 ± 0.25 ^a^	131.41 ± 0.24 ^e^	26.87 ± 0.15 ^d^	42.29 ± 0.34 ^c^	6.17 ± 0.00 ^a^	88.28 ± 0.48 ^b^
	600	35	90.22 ± 0.00 ^d^	58.69 ± 0.15 ^d^	130.73 ± 0.18 ^f^	31.53 ± 0.15 ^b^	40.52 ± 0.18 ^d e^	6.16 ± 0.01 ^a^	88.13 ± 0.28 ^b c^
		45	83.27 ± 0.25 ^f^	55.26 ± 0.15 ^e^	125.79 ± 0.21 ^g^	28.00 ± 0.10 ^c^	42.53 ± 0.46 ^c^	6.13 ± 0.03 ^a^	88.63 ± 0.03 ^b^
		55	72.46 ± 0.04 ^g^	50.43 ± 0.07 ^f^	116.21 ± 0.21 ^h^	22.03 ± 0.03 ^e^	43.75 ± 0.17 ^b^	6.16 ± 0.01 ^a^	88.48 ± 0.07 ^b^

Different small letters in the same items indicate a significant difference as determined by Duncan’s range test (*p* < 0.05), according to the treatment method.

## Data Availability

The data used to support the findings of this study can be made available by the corresponding author upon request.
